# Haemoglobin A_1c_ cut-off point to identify a high risk group of future diabetes: results from the Omiya MA Cohort Study

**DOI:** 10.1111/j.1464-5491.2012.03572.x

**Published:** 2012-07

**Authors:** M Kato, M Noda, H Suga, T Nakamura, M Matsumoto, Y Kanazawa, for the Omiya MA Cohort Study Group

**Affiliations:** 1 Japan Foundation for the Promotion of International Medical Research CooperationMiyahara-cho, Kita-ku, Saitama-shi, Saitama; 2 Department of Diabetes and Metabolic Medicine, and Diabetes Research Center, National Center for Global Health and MedicineMiyahara-cho, Kita-ku, Saitama-shi, Saitama; 3 Omiya Medical AssociationMiyahara-cho, Kita-ku, Saitama-shi, Saitama; 4 Japan Diabetes FoundationHongo, Bunkyo-ku, Tokyo, Japan

**Keywords:** diabetes, epidemiology, fasting blood glucose, HbA_1c_, impaired fasting glucose

## Abstract

**Aims:**

Using the HbA_1c_ level to define diabetes has several advantages and these advantages also apply to define a high-risk group. However, the risk of diabetes increases as HbA_1c_ increases and a certain degree of arbitrariness in the cut-off for the high risk group is unavoidable. The aim of this study was to determine the HbA_1c_ cut-off for defining a high-risk group that corresponds to the fasting plasma glucose cut-off by comparing the risk of diabetes against the fasting plasma glucose and HbA_1c_ levels in the Japanese population.

**Methods:**

A retrospective cohort study was conducted using data from annual health examinations performed in Omiya city. A total of 11 271 subjects between the ages of 40 and 79 years without diabetes at baseline were followed for up to 7 years. According to the new diagnostic criteria, diabetes was defined as an fasting plasma glucose level ≥ 7 mmol/l or an HbA_1c_ level ≥ 48 mmol/mol (≥ 6.5%) or a self-report. The HbA_1c_ cut-off corresponding to the fasting plasma glucose cut-off was determined using the incidence, hazard ratio, and a receiver operating characteristic analysis.

**Results:**

Eight hundred and sixty subjects developed diabetes. The incidence, hazard ratio, and receiver operating characteristic analysis all indicated that an HbA_1c_ cut-off of 39 mmol/mol (5.7%) corresponded to an fasting plasma glucose level of 5.6 mmol/l.

**Conclusions:**

Our results suggested that the HbA_1c_ cut-off for high-risk of diabetes should be 39 mmol/mol (5.7%), consistent with the American Diabetes Association recommendation. Further research is needed to determine whether our results are applicable to other populations.

## Introduction

Haemoglobin A_1c_ is a marker of cumulative glycaemic exposure over the preceding 2- to 3-month period and has been used as a monitoring tool for glycaemic control in diabetic patients. In January 2010, the American Diabetes Association (ADA) released a new definition for diabetes mellitus using an HbA_1c_ criterion (≥ 48 mmol/mol, ≥ 6.5%) in addition to the conventional fasting plasma glucose (FPG) criterion [1]. In July 2010, the Japan Diabetes Society also released a new definition of diabetes mellitus that included the HbA1c criterion [2]. Recently, the World Health Organization released an expert consultation report that accepted HbA_1c_ as an additional test for the diagnosis of diabetes. [3].

Using the HbA_1c_ level to define diabetes has several advantages over using the FPG level, such as the absence of the need to fast and a lower level of biological variability, and these advantages are also true for the definition of a high-risk group based on the HbA_1c_ level. The American Diabetes Association recommended an HbA_1c_ level of 39 mmol/mol (5.7%) [1] and the Japan Diabetes Society recommended a level of 38 mmol/mol (5.6%) [2] as the cut-off for a high-risk group. However, as the risk of diabetes increased as the HbA_1c_ level increased, a certain degree of arbitrariness in the cut-off point for defining a high-risk group is unavoidable. The aim of this study was to determine an HbA_1c_ cut-off value for defining a high-risk group that corresponds to the FPG cut-off value (5.6 mmol/l) [1,2] by comparing the risk of developing diabetes against the FPG and HbA_1c_ levels in a Japanese population.

## Participants and methods

This retrospective cohort study was conducted using anonymous data from annual health examinations performed in Omiya city by the Omiya Medical Association between 2000 (baseline) and 2007. The annual health examinations included a short questionnaire about medical condition and lifestyle. The questionnaire asked about the status (not present, under treatment, cured, or left untreated) of several medical conditions such as hypertension, cardiovascular disease, cancer, and diabetes.

Subjects who completed a health examination in 2000 were included in the present analysis if they were between the ages of 40 and 79 years and if their FPG and HbA_1c_ data were available at baseline (in 2000) (*n* = 24 694: 8103 men, 16 591 women). Subjects with missing baseline data (*n* = 6) and subjects with heart disease, stroke, chronic liver disease, kidney disease or any type of cancer at baseline (*n* = 3413) were excluded from the analysis. Because the present study examined the incidence of diabetes, health examination participants with diabetes at baseline (*n* = 1933) were also excluded. Subjects who did not undergo an annual health examination in 2001 (*n* = 8076) were subsequently excluded from the analysis because of the lack of follow-up data. Compared with the subjects who underwent an annual health examination in 2001, the subjects who did not undergo an annual health examination in 2001 were somewhat younger (mean age 61.2 years vs. 58.4 years), but no significant differences in their baseline FPG level (mean FPG, 5.2 mmol/l vs. 5.2 mmol/l) or HbA1c level [mean HbA1c 34 mmol/mol (5.3%) vs. 34 mmol/mol (5.3%)] were observed. The remaining cohort consisted of 11 271 subjects (3279 men and 7992 women).

Subjects were regarded as incident cases of diabetes if they became diabetic [FPG ≥ 7 mmol/l, HbA_1c_≥ 48 mmol/mol, (≥ 6.5%) or a response of ‘under treatment’, ‘cured’ or ‘left untreated’ to the question regarding diabetes status] for the first time during the course of the follow-up period. Subjects were regarded as censored cases if any of their annual health examination data was missing or their diabetes status was undetermined (missing FPG, HbA1c or questionnaire information) for the first time during the course of the follow-up period.

The HbA_1c_ concentration was measured at a central laboratory using high-performance liquid chromatography [HLC-723 G5 (from 2000 to 2002) and HLC-723 G7 (from 2003 to 2009); Tosoh Corporation, Tokyo, Japan] and calibrated using the standard calibrators of the Japan Diabetes Society. The Japan Diabetes Society value for HbA_1c_ can be transformed to a National Glycohemoglobin Standardization Program (NGSP) equivalent value by adding 0.4 to the Japan Diabetes Society value [2]; all the HbA_1c_ values in this manuscript were represented as the NGSP equivalent value. We used the HbA_1c_ threshold for the diagnosis of diabetes [48 mmol/mol (6.5%)] according to the new diagnostic criteria for diabetes adopted by the American Diabetes Association [1] and the Japan Diabetes Society [2].

To examine the association between FPG (or HbA_1c_) and the risk of future diabetes, we calculated the incidence of diabetes according to the baseline FPG (or HbA_1c_) level. To evaluate the risk of diabetes according to the FPG (or HbA1c) level, we calculated the hazard ratios adjusted for sex, age (categorized as 40–49, 50–59, 60–69 and 70–79 years), body mass index (categorized as < 19, 19–20.9, 21–22.9, 23–24.9, 25–26.9, 27–28.9 and ≥ 29 kg/m^2^), history of hypertension, family history of diabetes, alcohol intake (never, ex-drinker, occasional drinker and habitual drinker) and smoking status (never, ex-smoker and current smoker). As the data regarding diabetes was obtained at 1-year intervals, we treated the data as grouped survival time and analysed it using a complementary log-log regression model which corresponds to a proportional hazard model in continuous time cases [4].

## Results

**FIGURE 1:**
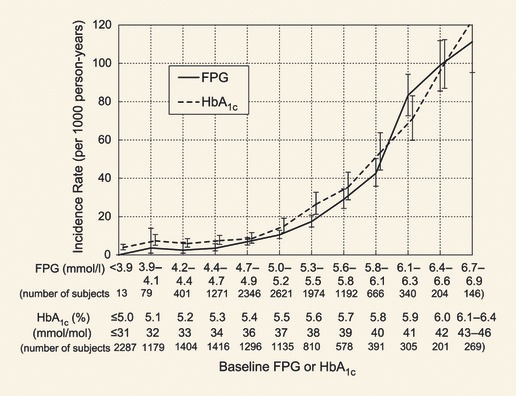
Comparison between fasting plasma glucose (FPG) and HbA_1c_ in terms of incidence rate. The FPG was divided into intervals with the same width [0.28 mmol/l (5mg/dl)]. The horizontal axes for HbA1c and FPG were placed so that the two curves overlapped.

The baseline characteristics of the subjects are shown in Table 1. At baseline, the proportion of subjects with an FPG level ≥ 5.6 mmol/l was 22.6% and the proportions of subjects with an HbA_1c_ level ≥ 38 mmol/mol (≥ 5.6%) and ≥ 39 mmol/mol (≥ 5.7%) were 22.7% and 15.5%, respectively. During the 7-year follow-up period (average follow-up period, 3.8 years), 860 subjects (354 men and 506 women) were identified as incident cases of diabetes by annual health checkups. Among the 860 incident cases, 394 cases were diagnosed according to the FPG criterion (FPG ≥ 7 mmol/l) and 443 cases were diagnosed according to the HbA_1c_ criterion [HbA_1c_ level ≥ 48 mmol/mol (≥ 6.5%)]. Among these incident cases, 110 cases were diagnosed using both the FPG and HbA_1_c criteria. The remaining 133 (= 860 – 394 – 443 + 110) cases were diagnosed according to the self-report (answered ‘under treatment’, ‘cured’, or ‘left untreated’ to the question regarding diabetes status) only. The incidence of diabetes increased as the baseline FPG or HbA_1c_ value increased and an almost similar pattern was observed irrespective of sex or age (see the Supporting Information, Fig. S1). The incidence according to the baseline FPG and HbA_1c_ values are shown together in Fig. 1. In Fig. 1, the horizontal axes for FPG and HbA_1c_ were placed so that the two curves for the incidence overlapped. The incidence (per 1000 person-years) for an FPG level of 5.6–5.8 mmol/l was 29.0 (95% CI, 24.3–34.5) and those for a HbA_1c_ level of 39 mmol/mol (5.7%) and 38 mmol/mol (5.6%) were 35.4 (95% CI 28.8–43.2) and 26.4 (95% CI 21.2–32.7), respectively. As shown in Fig. 1, an HbA_1c_ value of around 39 mmol/mol (5.7%) [between 38 mmol/mol (5.6%) and 39 mmol/mol (5.7%)] corresponded to an FPG level of 5.6 mmol/l.

**Table 1:** Baseline characteristics of subjects

	Total (*n* = 11 271)	Men (*n* = 3279)	Women (*n* = 7992)
Age (years)	62 (55–68)	65 (60–70)	61 (54–67)
BMI (kg/m^2^)	22.8 (2.9)	23.3 (2.7)	22.6 (3.0)
Fasting plasma glucose (mmol/l)	5.1 (4.8–5.5)	5.3 (4.9–5.6)	5.1 (4.8–5.4)
HbA_1c_ (%) HbA_1c_ (mmol/mol)	5.3 (5.1–5.5) 34 (32–37)	5.3 (5.1–5.5) 34 (32–37)	5.3 (5.1–5.5) 34 (32–37)
History of hypertension (yes)	71.4	66.3	73.5
Alcohol			
Never	52.6	20.1	65.9
Ex-drinker	1.0	2.2	0.6
Occasional-drinker	26.9	28.1	26.4
Habitual drinker	19.5	49.6	7.1
Smoking			
Never	76.5	42.4	90.6
Ex-smoker	9.0	25.2	2.3
Current smoker	14.5	32.5	7.1

Age, fasting plasma glucose and HbA_1c_ are represented as the median (interquartile range), BMI is represented as the mean (standard deviation); the other characteristics are represented as proportions.

**FIGURE 2:**
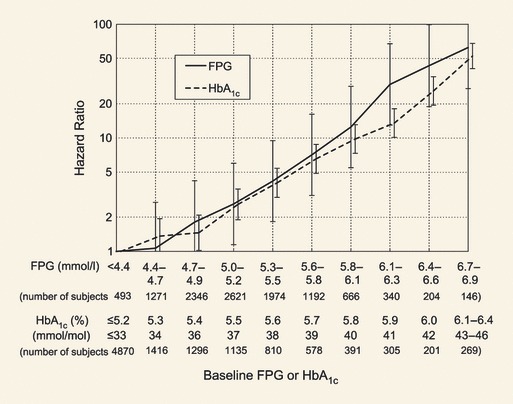
Comparison between fasting plasma glucose (FPG) and HbA_1c_ in terms of hazard ratio. The FPG was divided into intervals with the same width [0.28 mmol/l (5mg/dl)]. The horizontal axes for HbA_1c_ and FPG were placed so that the two curves overlapped. To calculate the hazard ratio, FPG levels < 4.4 mmol/l and an HbA_1c_ level ≤ 33 mmol/mol (≤ 5.2%) were combined into one category and used as the reference category, respectively.

The hazard ratios according to the baseline FPG or HbA1c values adjusted for sex, age, body mass index, history of hypertension, family history of diabetes, alcohol drinking status and smoking status are shown in the Supporting Information (Tables S1 and S2, respectively), and the hazard ratios are also shown together in Fig. 2. An FPG or HbA1c level with an almost constant incidence (< 4.4 mmol/l for FPG and ≤ 5.2% [≤ 33 mmol/mol] for HbA1c) was selected as a reference (see Figure 1). In Fig. 2, the horizontal axes for FPG and HbA1c were placed so that the two curves for the hazard ratios overlapped. The hazard ratio for an FPG level of 5.6 mmol/l was 7.08 (95% CI, 3.11–16.1) and that for an HbA1c level of 5.7% (39 mmol/mol) was 6.53 (95% CI, 4.87–8.75). As shown in Fig. 2, an HbA1c value of around 5.7% (39 mmol/mol) also corresponded to an FPG level of 5.6 mmol/l in terms of the hazard ratios.

**FIGURE 3:**
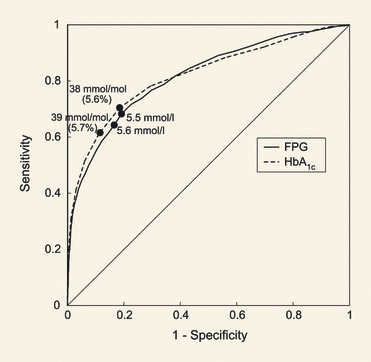
Receiver operating characteristic curve for development of diabetes against the baseline HbA_1c_ and fasting plasma glucose (FPG) levels.

The receiver operating characteristic curves for FPG and HbA_1c_ are shown in Fig. 3. The curves for FPG and HbA_1c_ are almost similar, indicating that FPG and HbA_1c_ have almost the same ability to detect future diabetes. The area under the curve values for FPG and HbA_1c_ were 0.82 (95% CI 0.80–0.83) and 0.82 (95% CI 0.80–0.84), respectively. The optimal cut-off values, which maximize the sum of the sensitivity plus specificity, were 5.5 mmol/l (sensitivity of 68% and specificity of 81%) and 5.6% (38 mmol/mol) (sensitivity of 70% and specificity of 81%), respectively. An FPG level of 5.6 mmol/l (sensitivity of 64% and specificity of 83%) and an HbA_1c_ level of 39 mmol/mol (5.7%) (sensitivity of 61% and specificity of 89%) were both adjacent to the optimal cut-offs and had a similar position on the receiver operating characteristic curves. In the receiver operating characteristic analysis, an FPG level of 5.6 mmol/l once again corresponded to an HbA_1c_ level of around 39 mmol/mol (5.7%) [between 38 mmol/mol (5.6%) and 39 mmol/mol (5.7%)[. The correspondence between an FPG level of 5.6 mmol/l and an HbA_1c_ level of 39 mmol/mol (5.7%) held true for both men and women.

## Discussion

For both the FPG and the HbA_1c_ levels, the risk of diabetes increased as the FPG or HbA_1c_ level increased, and no clear cut-off point exists above which the risk of diabetes increases markedly. Therefore, a certain degree of arbitrariness in the cut-off point for the high-risk group is unavoidable. In the case of the FPG level, the American Diabetes Association defined the cut-off point for the group with a high risk of developing diabetes (impaired fasting glucose) as 5.6 mmol/l [1,5], and this cut-off can also be applied to the Japanese population [6–8]. Thus, it seems reasonable to determine the HbA_1c_ cut-off point for the high-risk group in a manner such that the risk of the group defined by the cut-off point is similar to that of the high-risk group determined using the FPG cut-off. Our data for the incidence, hazard ratio and receiver operating characteristic analysis showed that an HbA_1c_ cut-off value of 39 mmol/mol (5.7%) corresponds to the FPG cut-off value of 5.6 mmol/l. When prevalence was considered, an FPG level of 5.6 mmol/l corresponded to an HbA_1c_ level of about 38 mmol/mol (5.6%) [between 38 mmol/mol (5.6%) and 39 mmol/mol (5.7%)], and the prevalence of individuals in the high-risk group defined by an HbA_1c_ level of 5.7% was smaller than that defined by the FPG level. Several cross-sectional studies have examined the correlation between FPG and HbA_1c_ and have found that an HbA_1c_ level around 5.6–5.7% appeared to be equivalent to an FPG level of 5.6 mmol/l [9,10]. In the present study, we also analysed the correlation between FPG and HbA_1c_, and an FPG level of 5.6 mmol/l and 5.8 mmol/l corresponded to an HbA_1c_ level of 38 mmol/mol (5.6%) and 39 mmol/mol (5.7%), respectively (data not shown). Taking into account that the FPG cut-off value of 5.6 mmol/l (100 mg/dl) must be a round number, we think that these results also support our conclusion.

Several papers have discussed the relationship between HbA_1c_ and the risk of developing diabetes [11–13], and some of these papers have been from Japan [14–16]. Based on these reports, the American Diabetes Association defined subjects with an HbA_1c_ level of between 39 mmol/mol (5.7%) and 46 mmol/mol (6.4%) as ‘categories of increased risk for diabetes’ [1]. Similarly, the Japan Diabetes Society defined subjects with an HbA_1c_ level of between 42 mmol/mol (6.0%) and 46 mmol/mol (6.4%) as ‘suspected diabetes mellitus cannot be excluded’ and between 38 mmol/mol (5.6%) and 39 mmol/mol (5.9%) as ‘a group with a high risk for developing diabetes mellitus in the future’ [2]. However, the analyses in the above-mentioned studies were based on categorized HbA_1c_ values and the proposed cut-off value was determined with some arbitrariness, as no clear cut-off point exists above which the risk of diabetes increases markedly. In this paper, the HbA_1c_ cut-off value was determined by comparing the risk of diabetes with the FPG cut-off level; to our knowledge, this is the first attempt to determine the cut-off value in this manner. We believe that this is a logical and reasonable way to define the cut-off value for HbA_1c_ and that it provides a solid basis for the above definition of the cut-off value of 39 mmol/mol (5.7%).

This study had several strengths. First, diabetes was defined using both FPG and HbA_1c_ according to the recent American Diabetes Association [1] and Japan Diabetes Society [2] diagnostic criteria for diabetes mellitus. Diagnosing diabetes based on the HbA_1c_ values is quite appealing, especially for epidemiological studies, because no glucose tolerance test or fasting blood sample is required. In addition, chronic hyperglycaemia, which is characteristic of diabetes mellitus, can be detected using a single measurement using HbA_1c_. Moreover, because the variability of HbA_1c_ is lower than that of FPG or the 2-h plasma glucose values [17–19], the potential risk for misclassification is also expected to be low. Second, the relatively large numbers of subjects and the long follow-up period of the present study make it possible to analyse the incidence using a relatively small HbA_1c_ interval. This is an important point because the correct identification of a high-risk group is only possible if a precise cut-off value is used. The present study also had several limitations. First, ‘diabetes’ in the present study was defined using a single measurement of FPG and of HbA1c. Defining diabetes using a single measurement of FPG may lead to an overestimation of the incidence of diabetes, as subjects with transient hyperglycaemia may be regarded as incident cases of diabetes. Although uncommon, subjects with a spuriously high HbA_1c_ level may also be incorrectly regarded as having diabetes. To investigate this point, we analysed the data by defining diabetes as an FPG ≥ 7 mmol/l and (not ‘or’) an HbA1c ≥ 48 mmol/mol (≥ 6.5%) in addition to the self-report. In this analysis, 515 of the 11 486 subjects developed diabetes, and although the incidence decreased, the correspondence between an FPG of 5.6 mmol/l and an HbA1c of 39 mmol/mol (5.7%) did not change. Second, although the diagnosis of diabetes based on the HbA_1c_ values has many advantages, several problems also exist. In addition to from the standardization problem, HbA_1c_ values do not reflect the plasma glucose level for subjects with abnormal haemoglobin or diseases that affect erythrocyte turnover, such as anaemia or liver cirrhosis [19–21]. However, these problems did not seem to be serious in the present study because (1) we excluded subjects with severe diseases, such as liver cirrhosis, and (2) diabetes was diagnosed based not only on the HbA_1c_ value, but also using the FPG level as well as self-report. Third, a relatively large number of subjects (about 42%) did not undergo an annual health check-up in 2001. This limitation arose from the study design, as the participants were allowed to decide whether they wished to undergo a health examination. Although the subjects who did not undergo an annual health examination in 2001 were younger than the subjects who underwent an annual health examination in 2001, no significant differences in the baseline FPG and HbA_1c_ levels were observed; consequently, a large bias was not thought to exist. Fourth, the subjects of the present study were participants of health check-ups and may not represent the general population. Generally, the participants of health check-ups are more health conscious than those who do not participate. However, whether the risk of diabetes, as determined using the FPG or HbA_1c_ levels exists between health check-up participants and non-participants remain unclear, and further research is needed to clarify this point.

Our study is one of several studies to reveal an association between HbA_1c_ and the future risk of diabetes in the Japanese population, and to determine the HbA_1c_ cut-off value for a high-risk group for future diabetes in not only a logical and reasonable but also a natural way, that is, by determining the HbA_1c_ cut-off value based on its correspondence with the FPG cut-off value according to the risk of developing diabetes.

## Supporting Information

**Figure S1** Incidence rate of diabetes mellitus according to baseline fasting plasma glucose (FPG) and HbA_1c_ levels.

FPG was divided into intervals with the same width (0.28 mmol/l [5mg/dl]).

**Table S1** Hazard ratios for the incidence of diabetes mellitus according to the baseline fasting plasma glucose (FPG) levels.

**Table S2** Hazard ratios for the incidence of diabetes mellitus according to the baseline HbA_1c_ levels.
