# Supplementary figures and images for: Haemoglobin A1c cut-off point to identify a high risk group of future diabetes: results from the Omiya MA Cohort Study
Source: Diabet Med. 2012 Jul;29(7):905–10. doi: 10.1111/j.1464-5491.2012.03572.x (PMC3504345; doi:10.1111/j.1464-5491.2012.03572.x)

Supporting Information

Figure S1

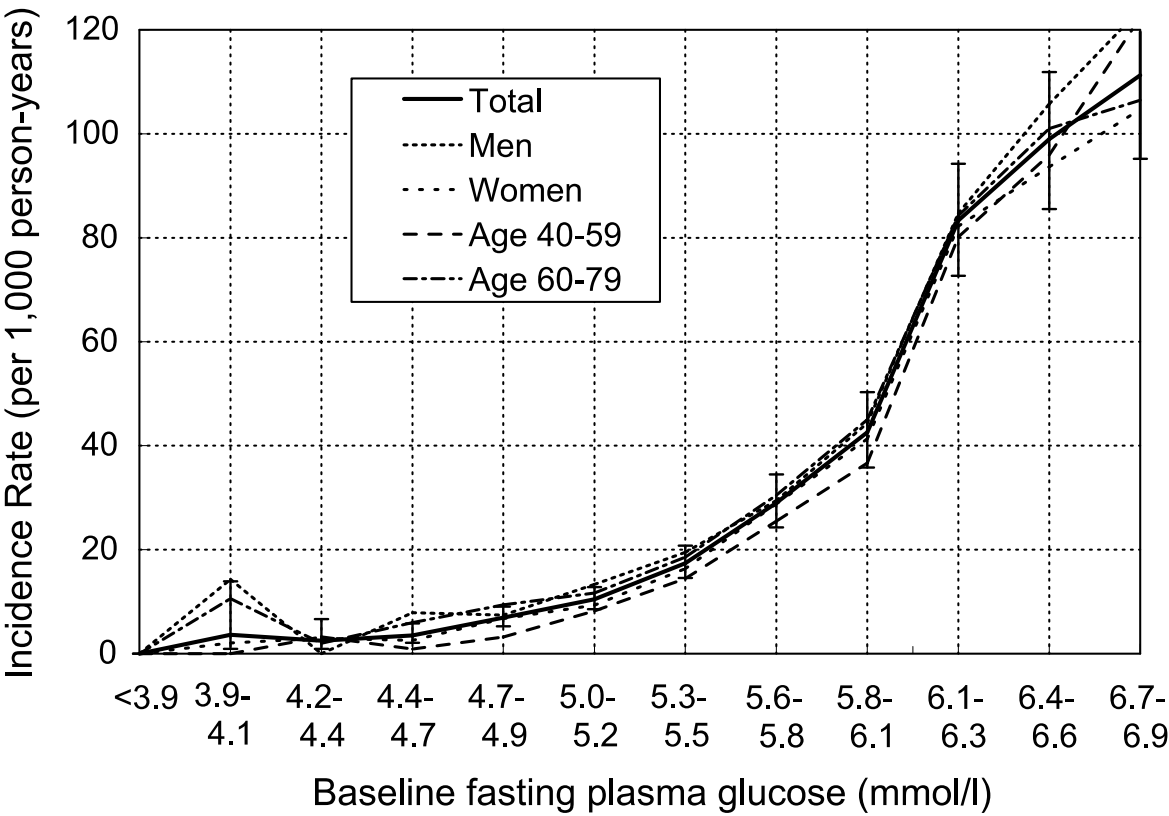

Supplement: Supplementary file 1 [file dme0029-0905-SD1.pdf]
