# Supplementary material for: Haemoglobin A1c cut-off point to identify a high risk group of future diabetes: results from the Omiya MA Cohort Study
Source: Diabet Med. 2012 Jul;29(7):905–10. doi: 10.1111/j.1464-5491.2012.03572.x (PMC3504345; doi:10.1111/j.1464-5491.2012.03572.x)
Supplement: Supplementary file 2 [file dme0029-0905-SD2.pdf]

Supporting Information

Table S1 Hazard ratios for the incidence of diabetes mellitus according to the baseline fasting plasma glucose (FPG) levels

	Total				Men				Women			
Baseline FPG (mmol/l)	*n*	Cases	Hazard ratio[*1]	95% CI	*n*	Cases	Hazard ratio[*2]	95% CI	*n*	Cases	Hazard ratio[*2]	95% CI
<4.4 (reference)	493	6	1		82	1	1		411	5	1	
4.4–4.7	1271	17	1.07	0.42–2.70	240	7	2.07	0.25–16.82	1031	10	0.80	0.27–2.33
4.7–4.9	2364	58	1.81	0.78–4.20	546	16	2.09	0.28–15.78	1818	42	1.74	0.69–4.40
5.0–5.2	2621	101	2.62	1.15–5.98	735	39	3.59	0.49–26.16	1886	62	2.27	0.91–5.67
5.3–5.5	1974	125	4.16	1.83–9.47	655	49	5.29	0.73–38.40	1319	76	3.76	1.52–9.33
5.6–5.8	1192	125	7.08	3.11–16.12	430	47	7.94	1.09–57.79	762	78	6.98	2.81–17.32
5.8–6.1	666	118	12.46	5.46–28.42	285	51	14.36	1.98–104.36	381	67	12.49	5.01–31.16
6.1–6.3	340	130	29.55	12.97–67.32	150	58	35.76	4.93–259.36	190	72	27.99	11.23–69.77
6.4–6.6	204	97	43.22	18.84–99.14	84	46	60.63	8.32–441.73	120	51	37.14	14.69–93.89
6.7–6.9	146	83	62.53	27.15–144.05	72	40	72.41	9.91–529.19	74	43	65.23	25.59–166.26

[*1]Adjusted for sex, age (40–49, 50–59, 60–69, and 70–79 years), body mass index (<19, 19–20.9, 21–22.9, 23–24.9, 25–26.9, 27–28.9 and ≥ 29 kg/m^2), history of hypertension, family history of diabetes (yes, no, and missing), alcohol intake (never, ex-drinker, occasional drinker, and habitual drinker) and smoking status (never, ex-smoker, and current smoker).

[*2]Adjusted for same variables (except for sex) as in *1.

Table S2 Hazard ratios for the incidence of diabetes mellitus according to the baseline HbA_{1c} levels

	Total				Men				Women			
Baseline HbA1c (%) (mmol/mol)	*n*	Cases	Hazard ratio*1	95% CI	*n*	Cases	Hazard ratio*2	95% CI	*n*	Cases	Hazard ratio*2	95% CI
≤5.2 (reference)	4870	101	1		1487	51	1		3383	50	1	
5.3 (34)	1416	44	1.36	0.96–1.94	411	18	1.19	0.70–2.05	1005	26	1.51	0.94–2.43
5.4 (36)	1296	43	1.46	1.02–2.09	325	22	1.87	1.13–3.09	971	21	1.21	0.72–2.01
5.5 (37)	1135	66	2.60	1.90–3.54	302	21	2.01	1.21–3.35	833	45	3.09	2.06–4.6
5.6 (38)	810	80	4.01	2.99–5.40	227	34	3.92	2.53–6.09	583	46	4.29	2.86–6.4
5.7 (39)	578	83	6.53	4.87–8.75	158	32	6.30	4.03–9.84	420	51	6.80	4.58–10.1
5.8 (40)	391	87	9.76	7.30–13.0	112	32	8.77	5.60–13.75	279	55	10.6	7.20–15.7
5.9 (41)	305	89	13.48	10.09–18.0	104	35	11.42	7.36–17.7	201	54	15.9	10.70–23.5
6.0 (42)	201	92	25.9	19.37–34.5	68	45	32.7	21.70–49.3	133	47	21.0	13.95–31.6
6.1–6.4 (43–46)	269	175	52.5	40.7–67.9	85	64	46.7	31.63–68.9	184	111	58.7	41.4–83.2

*1Adjusted for sex, age (40–49, 50–59, 60–69, and 70–79 years), body mass index (<19, 19–20.9, 21–22.9, 23–24.9, 25–26.9, 27–28.9 and ≥ 29 kg/m2), history of hypertension, family history of diabetes (yes, no, and missing), alcohol intake (never, ex-drinker, occasional drinker, and habitual drinker) and smoking status (never, ex-smoker, and current smoker).

*2Adjusted for same variables (except for sex) as in *1.
